# In Silico Analysis of Novel Bacterial Metabolites with Anticancer Activities

**DOI:** 10.3390/metabo14030163

**Published:** 2024-03-13

**Authors:** Pfariso Maumela, Mahloro Hope Serepa-Dlamini

**Affiliations:** Department of Biotechnology and Food Technology, Faculty of Science, University of Johannesburg, Doornfontein Campus, P.O. Box 17011, Johannesburg 2028, South Africa; hopes@uj.ac.za

**Keywords:** oligopeptides, anticancer, bacterial endophytes

## Abstract

Resistance to anticancer therapeutics is a major global concern. Thus, new anticancer agents should be aimed against novel protein targets to effectively mitigate the increased resistance. This study evaluated the potential of secondary metabolites from a bacterial endophyte, as new anticancer agents, against a novel protein target, fibroblast growth factor. In silico genomic characterization of the *Bacillus* sp. strain MHSD_37 was used to identify potential genes involved in encoding secondary metabolites with biological activity. The strain was also exposed to stress and liquid chromatography–mass spectrometry used for the identification and annotation of secondary metabolites of oligopeptide class with anticancer activity. Selected metabolites were evaluated for their anticancer activity through molecular docking and Absorption, Distribution, Metabolism, Excretion and Toxicity (ADMET) properties analysis. Phylogenetic analysis revealed that strain MHSD_37 shared close evolutionary relationships with *Bacillus* at the species level, with no identified relationships at the sub-species level. Both in silico genomic characterization and spectrometry analysis identified secondary metabolites with potential anticancer activity. Molecular docking analysis illustrated that the metabolites formed complexes with the target protein, fibroblast growth factor, which were stabilized by hydrogen bonds. Moreover, the ADMET analysis showed that the metabolites passed the toxicity test for use as a potential drug. Thereby, *Bacillus* sp. strain MHSD_37 is a potential novel strain with oligopeptide metabolites that can be used as new anticancer agents against novel protein targets.

## 1. Introduction

The significant global prevalence of cancer can be attributed to an increase in risk factors which expose humans to carcinogens. The main risk factors include smoking, obesity, and excessive alcohol consumption [1]. The risk factors subject normal cells to biological and chemical stress that introduce genetic changes and, consequently, proliferation and disruption of normal cell growth [2]. The number of new cancer cases was estimated at 18.1 million in 2020 [3]. Lung, female breast, and prostate cancer contributed to at least 40% of the total global cases [4]. Furthermore, approximately 2 million new cases and 600,000 cancer deaths were reported in the United States. Interestingly, lung cancer contributed to 21% of cancer deaths [5].

Cancer prevalence and deaths remain significantly high despite the availability of a range of therapies for managing the disease [3,4,5]. Cancer management interventions can be pharmacological or non-pharmacological [6]. Pain is a common symptom of cancer, and, thus, non-pharmacological strategies such as physical therapy, diet, and cold/hot therapy are used for pain management [7,8]. Chemotherapy, radiation therapy, and surgery are the principal strategies in pharmacological intervention [9,10,11]. Chemotherapy drugs, however, cause severe organ toxicity, thereby limiting their administration at higher doses [12]. Moreover, the occurrence of drug resistance severely impacts the effectiveness of cancer drugs [13,14]. Thus, knowledge about the mechanisms of anticancer therapy resistance is essential for the development of novel cancer therapeutic agents.

The major mechanisms of anticancer therapy resistance are drug efflux, mutation of drug targets, interference with apoptosis and DNA replication, and drug inactivation [15,16,17]. The modification of drug targets and interference with DNA replication are the two key strategies of drug resistance employed by cancer cells at the molecular level [15,18]; the former involves the overexpression or mutation of drug targets. The overexpression and gene amplification of the HER2-specific peptide have been identified as a resistance mechanism for the anti-HER2 agent, Trastuzumab [19]. Interference in DNA replication is a result of drug-topoisomerase-II-complex-induced DNA damage, during anticancer therapy [20].

Anti-apoptosis mediated anticancer therapy resistance is a result of the suppression of apoptosis in cancer cells [21]. The B-cell lymphoma/leukemia 2 (BCL-2) family of proteins has been linked with anti-apoptotic characteristics in tumor cells [22]. Drug efflux, detoxification, and inactivation are the main cancer therapy resistance strategies that develop at the initiation of or during the treatment stage [23,24]. Drug efflux is mediated through the expression of P-glycoprotein efflux pumps [25]. P-glycoprotein is an ATP-binding transporter, involved in the generation and maintenance of colorectal cancer drug resistance [26]. The transporter is capable of the rapid removal of anticancer drugs from target tumor cells [27]. Glutathione S-transferase has been reported to be important for the development of anticancer drug resistance through detoxification [28,29].

Furthermore, the development of new cancer therapies can be achieved by targeting factors involved in the pathophysiology of cancer [30]. New drug therapies can be targeted at inhibiting the development, growth, and the spread of cancer [31]. The drugs can be targeted towards the inhibition of angiogenesis, which is crucial for the development of blood vessel to supply cancer cells and support growth thereof [32]. Cancer cells release angiogenic factors, including transforming growth factor-β (TGF-β), angiogenin, vascular endothelial growth factor (VEGF), and fibroblast growth factor (FGF) [32,33,34]. These factors are responsible for initiating the proliferation, migration, and invasion of endothelial cells within new vascular structures [35]. Therefore, agents that target these factors will prevent the proliferation and migration of cancer cells and subsequent invasion of healthy cells.

DNA transcription is important for the growth, survival, invasion, metastasis, angiogenesis, and apoptosis of tumor cells [36,37,38]. Therefore, replication and transcriptional factors are potential targets of new cancer therapeutics [39]. The therapies can be targeted towards cyclic-dependent kinase and RNA polymerases [40]. Enzymes are another important group of potential targets for the development of new cancer therapeutics [41,42]. Aromatase enzyme is a potential target for breast cancer therapy. Aromatase is involved in the synthesis of estrogens which is responsible for the growth of breast cancer cells [43]. In addition, Protein kinase C is another key enzyme in tumor cells, with roles in the cell cycle, cell division, differentiation, and proliferation [44,45].

Therefore, this study will explore the potential of secondary metabolites, of bacterial endophyte origin, as anticancer agents. Bacterial endophytes are a rich source of secondary metabolites of biological importance due their existence in harsh environments [46]. Secondary metabolites were identified with liquid chromatography–mass spectrometry (LC–MS) following the exposure of the bacterial endophyte, *Bacillus* sp. strain MHSD_37, to stress. The metabolites identified were screened against FGF, using computational analysis, to gain insight into their therapeutic potential as new anticancer agents.

## 2. Materials and Methods

### 2.1. Plant Material Collection and Bacterial Endophyte Isolate

Leaves from *Solanum nigrum*, a medicinal plant, were collected from a sandy location in Botlokwa, Ga-Ramatšowe, Limpopo Province, South Africa (−23.491054, 29.746048), in March 2017. The leaves were stored in a sterile polyethylene bag and transported to the laboratory at a temperature of 4 °C. The identification of the plant material was performed at the University of Johannesburg Herbarium (JRAU) and a sample of the plant material specimen was subsequently deposited in JRAU with voucher specimen number Serepa-Dlamini 209 and species name *Solanum nigrum*. The remainder of the leaves were immediately processed in the laboratory and the bacterial endophytes were isolated sequentially by washing the leaves in water for 1 min, 70% ethanol for 1.5 min, 1% sodium hypochlorite (NaOCl, Merck, Saint Louis, MO, USA) for 3 min, and a final wash in sterile distilled water three times. The water from the final wash was plated as a negative control. The surface sterilized leaves were ground in 2 mL of saline using a sterile pestle and mortar and the resultant homogenate was streaked onto nutrient agar (NA, Becton Dickson, Franklin Lakes, NJ, USA) plates under sterile conditions. Bacterial growth was monitored daily after incubating the plates for 24–48 h at 28 °C. The grown colonies were re-cultured three times in NA and under the conditions mentioned above to get pure colonies with uniform morphology. A 30% glycerol (glycerol diluted in sterile distilled water (*v*/*v*), Merck, Saint Louis, MO, USA) stock culture was prepared for each bacterial endophyte and stored at −80 °C for future use.

### 2.2. Bacterial Strain Maintenance

A 30% glycerol (Merck, Saint Louis, MO, USA) stock of the bacterial cultures was plated on NA (Becton Dickson, Franklin Lakes, NJ, USA) plates and incubated for 24 h at 28 °C for routine culture maintenance. The bacteria were grown on nutrient broth (NB, Becton Dickson, Franklin Lakes, NJ, USA) at 28 °C, agitating at 150 rpm for 24 h.

### 2.3. Genome Extraction, Library Preparation, and Sequencing

The NucleoSpin microbial DNA extraction kit (Macherey-Nagel, Düren, Germany) was used to extract the genomic DNA from the solid colonies as per the manufacturer’s protocol. The DNA was sequenced at the Biotechnology Platform, Agricultural Research Council, Onderstepoort, South Africa, a commercial service provider. Paired-end libraries (2 × 150 bp) were generated using the MGIEasy Universal DNA Library preparation kit (MGI Tech Co., Guangdong, China), and the sequencing was performed on the MGIEasy^®^ platform.

### 2.4. Genome Assembly and Annotation

Genome quality control, trimming, and assembly were performed on GALAXY, accessible from https://usegalaxy.org/ (accessed on 15 July 2023) [47]. FastQC (v 0.72.0) was used for the quality control of raw sequence reads which were then trimmed with the Trimmomatic program (version 0.38.0) [48]. The reads were de novo assembled using Unicycler (v 0.4.8.0) [49], and the quality was assessed with Quast (Galaxy v 5.0.2) [50]. The draft genome was annotated using the National Center for Biotechnology Information—Prokaryotic Genome Annotation Pipeline (PGAP) [51] and Rapid Annotations using Subsystems Technology (RAST accessed on 21 July 2023) [52].

### 2.5. Phylogenetic Analysis

A whole genome-based taxonomic analysis was done using a free bioinformatics platform, Type Strain Genome Server (TYGS), accessible from https://tygs.dsmz.de (accessed on 21 July 2023) [53]. A pairwise comparison among a set of genomes was performed with the Genome Blast Distance Phylogeny, and accurate intergenomic distances were inferred under the algorithm trimming and distance formula d2 [54]. The average nucleotide identity (ANI) values between the strain and closely related species were calculated with Orthologous Average Nucleotide Identity Tool (OAT) software (Version 0.90) [55]. The Genome-to-Genome Distance Calculator was accessed from https://ggdc.dsmz.de/ (accessed on 21 July 2023) [53].

### 2.6. Liquid Chromatography–Mass Spectrometry Analysis (LC–MS)

The bacterial excretome, following exposure to lead (Pb), was analyzed with a liquid chromatography-quadrupole time-of-flight tandem mass spectrometer (LC–MS-9030 q-TOF, Shimadzu Corporation, Kyoto, Japan) fitted with a Shim-pack Velox C18 column (100 mm × 2.1 mm with particle size of 2.7 m). The column oven temperature was maintained at 50 °C. The injection volume was 5 µL, and the samples were analytically separated over a 30 min binary gradient. A constant flow rate of 0.04 mL/min was applied using a binary solvent mixture of water with 0.1% formic acid (Eluent AMerck, Saint Louis, MO, USA) and 0.1% formic acid in acetonitrile (Eluent B, Merck, Saint Louis, MO, USA). The gradient technique was gradually increased from 3 to 30 min to facilitate the separation of the compounds within the samples. Eluent B was kept at 5% from 0 to 3 min, gradually increased from 5 to 40% between 3 and 5 min, and finally increased to 40–95% between 5- and 23-min. Eluent B was subsequently kept isocratic at 95% between 23 and 25 min. The gradient was returned to original conditions of 5% at 25–27 min, and re-equilibration at 5% occurred at 27–30 min. The liquid chromatographic eluents were subsequently subjected to a Quadruple Time-of-Flight high-definition mass spectrometer for analysis in positive electrospray ionization (ESI) mode with the following conditions: 400 °C heat block temperature, 250 °C Desolvation Line (DL) temperature, 42 °C flight tube temperature, and 3 L/min nebulization and dry gas flow. The data was acquired using the Data-dependent acquisition (DDA) mode, which simultaneously generated MS1 and MS2 data for all ions within a mass-to-charge ratio (*m*/*z*) range of 100–1500 (precursor m/z isolation window) and an intensity threshold above 5000. The MS2 Experiments were conducted utilizing argon gas as the collision gas and a collision energy of 35 eV with a spread of 5 and sodium iodide (Merck, Saint Louis, MO, USA) as a calibration solution to monitor high mass precision. Metabolite annotation was completed at Metabolomics Standards Initiative (MSI) levels 2 and 3. The former is based on the retention time, mass-to-charge ratio (*m*/*z*), and fragmentation patterns matching data available from the databases in Sirius [56,57,58]. The fragments with no matches to anything on the databases were classified according to their compound class according to the molecular networking from Canopus on Sirius [57,58]. Literature data was subsequently used to identify the biological activities of the annotated metabolites.

### 2.7. Secondary Metabolites and Proteins Selection

Four secondary metabolites belonging to the oligopeptides class were selected based on their potential identified anticancer activity, from literature data, and lack of an associated patent. The simple data forma (SDF) files for the 3-D structures of the selected metabolites of the oligopeptide class were retrieved from PubChem (https://pubchem.ncbi.nlm.nih.gov/, accessed on 18 December 2023) and named metabolite 1–4, and their PubChem IDs were also used to differentiate among the 4 metabolites. The collected structures of the metabolites were further optimized using Avogadro. The crystallography structure of the FGF protein target (PDB: 1CVS) was retrieved from the protein data bank (PDB) database (www.rcsb.org, accessed on 18 December 2023).

### 2.8. Molecular Docking

Molecular docking studies were carried out to estimate the binding energies of the metabolites towards the therapeutic protein targets, FGF, by using the computational program AutoDock Vina 1.1.2 [59]. The protein’s 3-D structure was modelled using ChimeraX _(Version 1.7) to generate a fine structure. The non-standard residues from the target protein were removed. The SDF and PDB files of the metabolites and the target protein were converted into a PDBQT format with the AutoDock (Version 1.5.7) tools. A grid box was created for the random docking of the protein. The grid box dimensions, spacing, and the grid map coordinate center for the random docking of the target protein were 60 Å × 60 Å × 60 Å, 0.375 Å, and x = 67.596, y = 23.207, and z = 118.874, respectively. Molecular docking analysis was performed with the Lamarckian genetic algorithm (LGA), and the docked structures were analyzed by using ChimeraX.

### 2.9. Absorption, Distribution, Metabolism, Elimination, and Toxicity (ADMET) Analysis

The four metabolites used in the dock studies were screened for their absorption, distribution, metabolism, elimination, and toxicity (ADMET) using the online tool http://biosig.unimelb.edu.au/pkcsm/prediction (accessed on 3 January 2024) to predict their important pharmacokinetic properties. The ADMET properties included absorption, human intestinal absorption, water solubility, Caco-2 permeability, P-glycoprotein substrate, P-glycoprotein I and II inhibitors, skin permeability, and distribution: steady state volume of distribution (VDss), fraction unbound, blood-brain barrier (BBB) permeability, and minnow toxicity [60].

## 3. Results

### 3.1. Phylogeny Characterisation of Strain MHSD_37 and Secondary Metabolite Biosynthesis Gene Clusters Analysis

**Figure 1:**
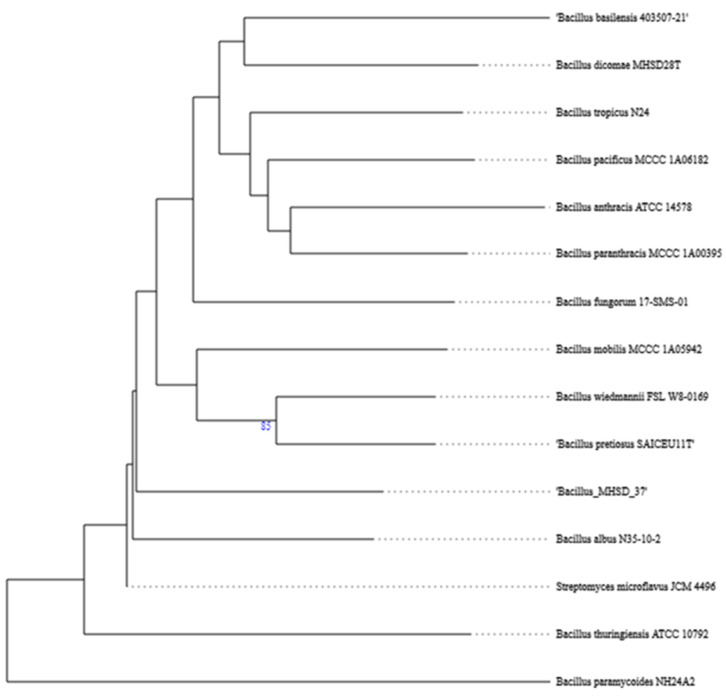
Phylogenetic tree illustrating the evolutionary relationships of strain MHSD_37.

**Figure 2:**
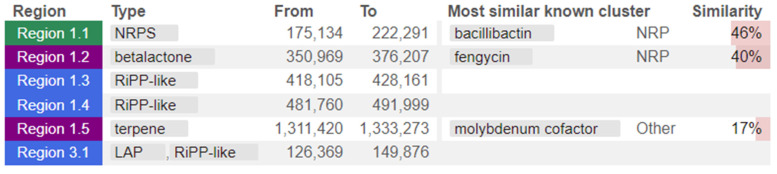
Strain MHSD_37 genome annotated regions encoding for secondary metabolites. The colors illustrate regions from the different class of secondary metabolites.

The whole genome of strain MHSD_37 was subjected to taxonomic analysis to determine the strain’s evolutionary descendancy. The evolutionary relationships of strain MHSD_37 are illustrated in the evolutionary tree in Figure 1. MHSD_37 shares the most recent common ancestor with a range of *Bacillus* sp. and is closely related to *Bacillus albus* N35-10-2 at the species level, thus it had the highest digital DNA–DNA hybridization (dDDH) with the strain. The strain was, however, not closely related to any of the *Bacilli* at the sub-species level, according to the evolutionary tree, which implies that strain MHSD_37 observed ANI values were below the species boundary value (ANI > 95–96%). Thus, strain MHSD_37 is a potential novel *Bacillus* sp. The antiSMASH analysis identified three regions which encode for the synthesis of secondary metabolites (Figure 2). Two of the regions encoded for metabolites with at least 40% similarity to bacillibactin and fengycin.

### 3.2. Identification and Annotation of Secondary Metabolites Using LC–MS

The untargeted metabolomics approach was applied to identifying the range of secondary metabolites synthesized and secreted by strain MHSD_37 when exposed to stress in the form of the toxic heavy metal Pb. Table 1 is a summary of biological active secondary metabolites selected from the global view profiled through untargeted metabolomics. Strain MHSD_37 synthesized secondary metabolites with antimalarial, antiviral, antibacterial, and anticancer activity (Table 1). The annotated metabolites belong to the classes: diterpenoids, terpene glycosides, alpha amino acids, and oligopeptides. Interestingly, the strain synthesized a notable number of oligopeptides with anticancer activity (Table 1).

### 3.3. In Silico Analysis of the Anticancer Potential of Secondary Metabolites from MHSD_37

**Figure 3:**
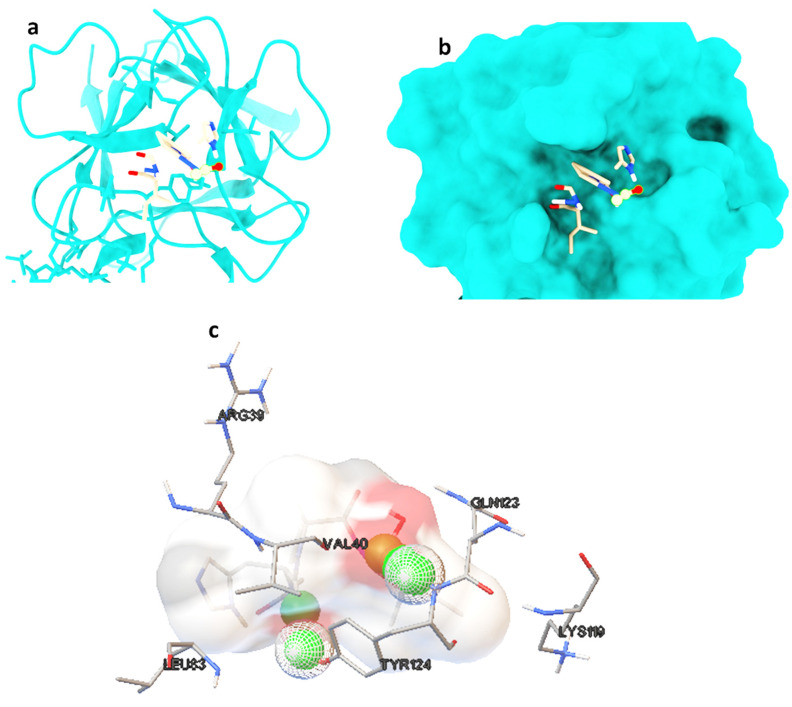
The best ligand-target protein complex (**a**), ligand docked pose of metabolite 1 with FGF (**b**), and hydrogen bond between the ligand and FGF in the best conformation (**c**). The hydrogen bonds are represented by the green balls.

**Figure 4:**
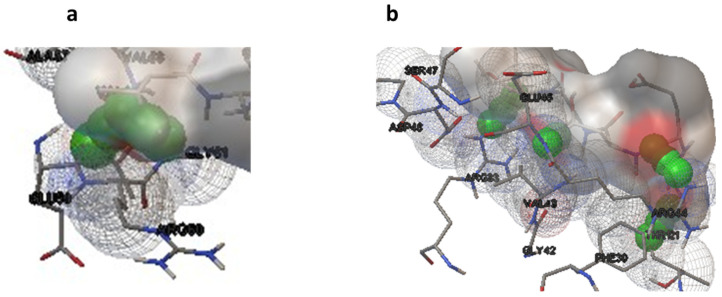
Hydrogen bonds for the best conformation of metabolite 4 (**a**) and 2 (**b**). The hydrogen bonds are represented by the green balls.

Molecular docking studies were performed on selected oligopeptides identified to have potential anticancer but with no known annotated identity or patent. A total of four oligopeptides were identified to have potential anticancer activity (Table 1). Their 3-D structures were obtained from PubChem and subsequently used for screening against the protein target, FGF. The screening was achieved through random molecular docking using the computational program AutoDock. Table 2 is a summary of the docking scores of the metabolites and their important molecular interactions with FGF. The metabolites formed molecular interactions dominated by hydrogen bonds to stabilize the interaction complex with the protein target. Metabolite 1 and 3 had the highest binding energy of −3.13 and −3.46 kcal/mol (Table 2), respectively. Figure 3 is an illustration of a 3-D best complex between the ligand and FGF (Figure 3a), docked pose of the ligand with FGF (Figure 3b), and hydrogen bond between the ligand and FGF in the best conformation (Figure 3c). Metabolite 1 formed a hydrogen bond with TYR 124 (TYR 124:HN and TYR 124:HH) as illustrated in Figure 3c. Metabolite 3 was also stabilized by a single hydrogen bond, LEU 98:HN, at its best conformation (Figure 3c). Metabolite 4 had the lowest binding energy of −1.06 kcal/mol (Table 2). The metabolite had two hydrogen bonds, THR 1:H1 and GLY 61:HN, with the protein target FGF (Figure 4a). Metabolite 2, which had the second lowest binding energy (−1.34 kcal/mol), had three hydrogen bonds, ARG 33:H, ARG33:HH1, and GLU 45:HN, with the protein target (Figure 4b).

### 3.4. ADMET Screening of the Secondary Metabolites

The metabolites were further screened for their therapeutic suitability, based on their pharmacokinetic profiles and toxicity properties, using in silico ADMET analysis. Table 3 is a summary of the ADMET properties for the four secondary metabolites. All four metabolites had an intestinal absorption of less than 30%, with metabolite 1 having the highest absorption of 27% (Table 3). The metabolites also had low Caco 2 permeability, ranging between −2.811 and −3.491. The predicted *T. pyriformis* toxicity was 0.285 log mM for all four metabolites. The minnow toxicity ranged between 3.444 and 7.633 log mM for the metabolites (Table 3).

## 4. Discussion

Cancer remains a prevalent disease and predominant cause of death globally, despite the availability of a wide array of therapies [3,4,5]. Cancer cells have developed resistant mechanisms which have had a significant impact on the effectiveness of cancer therapeutics [14,15,16,17]. New cancer therapeutics should, thus, be focused on novel protein targets. A case in point is FGF, because of the protein’s involvement in tumor cell growth and spread [35]. On the other hand, bacterial endophytes are a rich source of biologically active secondary metabolites with potential anticancer activity [58,59]. Bacterial endophytes thus are an important source in the continued search of new cancer therapeutics targeting novel target proteins. Furthermore, bioinformatics and computational biology offer cost effective and efficient approaches for the screening of novel anticancer agents.

This study applied whole genome sequencing and assembly to identify and characterize a bacterial endophyte isolated from the leaves of *Solanum nigrum*. The plant is widely found in wastelands due to its resilience and is used in traditional medicine for the treatment of tumors, asthma, inflammation, bacterial, and viral infections [90]. *De novo* sequencing and assembly provide researchers with tools to identify and characterize bacteria at the sub-species level [91]. The isolated strain was a *Bacillus* species with endophytic characteristics. The strain was, however, not closely related to any known *Bacillus* at the sub-species level, thereby illustrating that it is a potentially novel subspecies of *Bacillus* [92].

The genome annotation from antiSmash identified three regions encoding for secondary metabolites. Two of the regions encoded for secondary metabolites with a significant similarity to bacillibactin and fengycin, which have been reportedly found to be predominant in *Bacillus* [86,87,93]. The former is a siderophore with antimicrobial activity and is also widely applied as a biocontrol agent [93]. Fengycin is an antimicrobial lipopeptide, has been shown to have fungicidal activity, and has potential in agricultural applications [54]. Bacterial endophytes have been shown to produce bacillibactin and fengycin as defense mechanisms against competing microbes, thereby enabling them to maintain their dominance and establish a symbiotic relationship with the host [86,93].

The LC–MS analysis further established the existence of a wide range of secondary metabolites. The metabolites of oligopeptide class were identified as having potential anticancer activity. Oligopeptides have been successfully evaluated for their anticancer activity [94,95]. An oligopeptide isolated from *Anthopleura anjunae* was reported to induce apoptosis in prostate cancer cells, with the apoptotic cells showing an increase in Pro-apoptotic proteins, such as Bax, cytochrome-C, caspase-3, and caspase-9 [94]. A cationic peptide was reported to have dual potential as a drug delivery system for targeted therapy and an anticancer therapeutic agent [95].

The protein ligand interactions illustrated that the metabolites and the target protein, FGF, formed hydrogen bonds to stabilize the protein-ligand complexes. The binding energies of the complexes ranged between −1.06 and −3.46 kcal/mol. Binding affinities of between −3.51 and −8.42 Kcal/mol have been reported for metabolites from *Ficus carica*, with a range of anticancer target proteins [96]. In addition to efficacy, safety is also an important factor for successful drug development; therefore, ADMET properties analysis is crucial during drug discovery and development [97]. Although the secondary metabolites from MHSD_37 had poor intestinal solubility and Caco 2 permeability, they showed low toxicity. The poor solubilities are, however, not a major hindrance because the most important and challenging issue is the delivery to the tumor site and the maintenance of low toxicity. Furthermore, more emphasis should be placed on enhancing the production cost, selectivity, and proteolytic stability [98].

## 5. Conclusions

The bacterial endophyte, *Bacillus* sp. strain MHSD_37 is a rich source of secondary metabolites with a range of biological activities including anticancer, antimicrobial, and antiviral activities. The strain synthesizes metabolites of the oligopeptide class with potential anticancer activity. Molecular docking studies, based on oligopeptides with no patents, showed that the strain synthesized oligopeptides which could target the FGF protein, an important growth factor involved in anticancer drug resistance. Moreover, the ADMET analysis illustrated that the oligopeptides had low toxicity. Therefore, strain MHSD-37 is a promising source of secondary metabolites for the development of novel anticancer agents.

## Figures and Tables

**Table 1:** Annotated secondary metabolites identified from the excretome of strain MHSD_37.

Precursor (*m*/*z*)	Retention Time	Fragments	Class	Molecular Formula	Metabolite Annotation	Biological Activity	References
734.31	6.13	716, 690, 672, 660	Diterpenoids	C_40_H_47_NO_12_	3′-N-Debenzoyl-2′-deoxytaxol	Anticancer	[61]
545.26	6.4	412, 242, 155	Oligopeptide	C_22_H_36_N_6_O_10_	Acetyl-DTTPA-NH2	Anti-HIV	[62]
261.12	6.73	188, 136, 107	Alpha amino acid	C_14_H_16_N_2_O_3_	Maculosin	Antioxidant	[63]
314.17	7	215	Lipid	C_15_H_22_O_3_	Racemosalactone A	Anticancer	[64]
197.13	7.01	154, 112	Alpha amino acid	C_10_H_16_N_2_O_2_	Cyclo(-Pro-Val)	Antifungal	[65]
528.27	7.31	510, 464, 286, 299	Oligopeptide	C_23_H_37_N_5_O_9_	n.a.	Antimalarial	[66]
262.14	7.39	120, 116, 106	Peptide	C_14_H_18_N_2_O_3_	Phenylalanylproline	Antimicrobial	[67]
530.25	7.61	318, 300, 205, 171, 143	Oligopeptide	C_22_H_35_N_5_O_10_	n.a.	Anticancer	[68]
765.34	7.66	652, 608, 579, 466, 419	Oligopeptide	C_38_H_48_N_6_O_11_	n.a.	Antimalarial	[69]
408.23	7.69	293, 235, 156, 128, 109	Oligopeptide	C_19_H_29_N_5_O_5_	n.a.	Anti-angiotensin II	[70]
680.37	7.69	549, 452, 434, 424, 406	Oligopeptide	C_31_H_49_N_7_O_10_	n.a.	Anticancer	[71]
702.35	7.69	658, 575, 545, 462	Oligopeptide	C_36_H_49_N_5_O_8_	n.a.	Anti-virus	[72]
401.21	7.76	286, 258, 173, 171, 143	Oligopeptide	C_17_H_28_N_4_O_7_	n.a.	Antibacterial	[73]
444.23	7.77	369, 301, 237, 186, 141	Oligopeptide	C_16_H_29_N_9_O_6_	n.a.	Anticlot	[74]
888.42	7.79	863, 757, 747, 677, 653	Oligopeptide	C_39_H_59_N_11_O_14_	n.a.	Anti-virus	[75]
587.31	8.25	438, 411, 417, 354, 343	Oligopeptide	C_25_H_42_N_6_O_10_	n.a.	Antimicrobial	[76]
757.32	8.29	658, 583, 511, 485, 468	Oligopeptide	C_38_H_48_N_2_O_14_	n.a.	Anticancer	[77]
411.26	8.32	298, 215, 197, 181, 169	Oligopeptide	C_20_H_34_N_4_O_5_	n.a.	Antimicrobial	[67]
211.14	8.37	183, 154, 138, 114	Alpha amino acid	C_11_H_18_N_2_O_2_	Gancidin W	Antimalarial agent	[78]
578.29	8.39	447, 417, 402, 384, 316	Oligopeptide	C_27_H_39_N_5_O_9_	n.a.	Antimalarial agent	[68]
574.32	9.17	505, 461, 344, 314, 243	Oligopeptide	C_29_H_43_N_5_O_7_	n.a.	Anti-virus	[79]
701.32	9.29	536, 518, 477, 449, 423	Phenylalanine	C_38_H_44_N_4_O_9_	n.a.	Anti-virus	[80]
481.21	9.48	384, 338, 237	Oligopeptide	C_25_H_28_N_4_O_6_	n.a.	Anticancer	[81]
883.27	9.88	690, 672, 611, 589, 536	Cyclic depsipeptides	C_39_H_42_N_6_O_18_	Corneybactin	Iron acquisition	[82]
365.28	14.78	307, 287, 262, 240, 126	Alpha amino acids	C_19_H_39_N_2_O_3_	Empigen BR	Surfactant	[83]
279.16	16.51	149, 140, 121	Benzoic acid esters	C_16_H_22_O_4_	Hatcol DBP	Plasticizer	[84]
362.21	19.11	232, 203, 176, 105	Cinnamic acid esters	C2_4_H_27_NO_2_	Octocrylene	Sunscreen	[85]
631.41	24.44	599, 585, 379, 333, 323	Terpene glycosides	C_33_H_58_O_11_	Kurilensoside f	Antimicrobial	[86]
506.53	24.48	268, 258, 239	N-acyl amines	C_34_H_67_NO	Oleyl palmitamide	Plasticizer	[87]
551.59	24.48	506, 297, 268, 107	N-acyl amines	C_36_H_74_N_2_O	Butanamide, 4-(dioctylamino)	Anticancer	[88]
547.4	25.53	323, 305, 193, 165	Benzoic acid esters	C_33_H_54_O_6_	hatcol 2000	Plasticizer	[89]

**Table 2:** Binding energies and important interactions for the best complex of metabolites.

Metabolite	PubChem ID	Binding Energy (kcal/mole)	Important Interactions
1	44420768	−3.13	Hydrogen bond: TYR124:HN, TYR124:HH.
2	126672973	−1.34	Hydrogen bond: ARG33:HH11; ARG44:HH11, GLU45:HN
3	12376189	−3.46	LEU98:HN
4	102173172	−1.06	THR1:H1, GLY61:HN

**Table 3:** ADMET properties for the metabolites from strain MHSD_37 with potential anticancer activity against FGF.

	Water Solubility	Caco2 Permeability	Intestinal Absorption (Human)	Skin Permeability	P-Glycoprotein Substrate	P-Glycoprotein I Inhibitor	P-Glycoprotein II Inhibitor	VDss (Human)	Fraction Unbound (Human)	BBB Permeability	*T. Pyriformis* Toxicity	Minnow Toxicity
	Numeric (log mol/L)	Numeric (log Papp in 10^−6^ cm/s)	Numeric (% Absorbed)	Numeric (log Kp)	Categorical (Yes/No)	Categorical (Yes/No)	Categorical (Yes/No)	Numeric (log L/kg)	Numeric (Fu)	Numeric (log BB)	Numeric (log μg/L)	Numeric (log mM)
3	−2.811	−0.837	13	−2.375	Yes	No	No	−0.617	0.457	−1.467	0.285	3.444
1	−2.872	−0.517	27	−2.375	Yes	No	No	−0.899	0.684	−1.48	0.285	3.828
2	−2.85	−0.813	0	−2.735	Yes	No	No	−1.191	0.543	−1.566	0.285	8.151
4	−3.49	−0.307	17	−2.37	Yes	No	No	−1.21	0.464	0.065	0.285	7.633

## Data Availability

The data from the Whole Genome Shotgun project has been deposited at DDBJ/ENA/GenBank under the accession JAVBIS000000000, BioSample accession number SAMN36845528, and BioProject accession number PRJNA1002565. The version described in this paper is JAVBIS000000000.
